# A between-herd data-driven stochastic model to explore the spatio-temporal spread of hepatitis E virus in the French pig production network

**DOI:** 10.1371/journal.pone.0230257

**Published:** 2020-07-13

**Authors:** Morgane Salines, Mathieu Andraud, Nicolas Rose, Stefan Widgren

**Affiliations:** 1 ANSES, French Agency for Food, Environmental and Occupational Health & Safety, Ploufragan-Plouzané-Niort Laboratory, Epidemiology, Health and Welfare Research Unit, France; 2 Department of Disease Control and Epidemiology, National Veterinary Institute, Sweden; Universite Claude Bernard Lyon 1, FRANCE

## Abstract

Hepatitis E virus is a zoonotic pathogen for which pigs are recognized as the major reservoir in industrialised countries. A multiscale model was developed to assess the HEV transmission and persistence pattern in the pig production sector through an integrative approach taking into account within-farm dynamics and animal movements based on actual data. Within-farm dynamics included both demographic and epidemiological processes. Direct contact and environmental transmission routes were considered along with the possible co-infection with immunomodulating viruses (IMVs) known to modify HEV infection dynamics. Movements were limited to 3,017 herds forming the largest community on the swine commercial network in France and data from the national pig movement database were used to build the contact matrix. Between-herd transmission was modelled by coupling within-herd and network dynamics using the SimInf package. Different introduction scenarios were tested as well as a decrease in the prevalence of IMV-infected farms. After introduction of a single infected gilt, the model showed that the transmission pathway as well as the prevalence of HEV-infected pigs at slaughter age were affected by the type of the index farm, the health status of the population and the type of the infected farms. These outcomes could help design HEV control strategies at a territorial scale based on the assessment of the farms’ and network’s risk.

## 1. Introduction

Hepatitis E virus (HEV) is a non-enveloped single-stranded RNA virus frequently leading to asymptomatic infections in humans, but also causing acute or chronic hepatitis—depending, inter alia, on the patient’s immune status [1, 2]. If genotypes 1 and 2 are exclusively human viruses mainly present in developing countries, genotypes 3 and 4 are shared by humans and other animal species and are responsible for sporadic human cases in industrialised countries [3, 4]. In particular, HEV-3 is highly prevalent in European swine populations [5], e.g. in the French pig production sector, where around 65% of farms have been found to host at least one HEV seropositive pig [6]. A number of locally acquired cases have been linked to the consumption of raw or undercooked pork products, especially those containing liver in high proportion [7–16]. In that way, hepatitis E is recognised as a foodborne zoonosis with domestic pigs being the major reservoir in Western countries [17].

The risk of slaughtering HEV-positive pigs, and thus to enter contaminated products into the food chain, is strongly related to HEV dynamics in pig herds. Observational and experimental studies have evidenced several risk factors affecting HEV behaviour on pig farms, such as husbandry practices in terms of hygiene, biosecurity and rearing conditions [18], piglet’s sex and sow’s parity [19]. The protection conferred by maternally-derived antibodies (MDAs) was also shown to impact HEV dynamics [20, 21]. Moreover, pigs exhibited chronic hepatitis when co-infected with immunomodulating viruses (IMVs), e.g. porcine reproductive and respiratory syndrome virus (PRRSV) or porcine circovirus type 2 (PCV2) [19, 22, 23]. Recently, we have developed a stochastic individual-based model representing HEV spread and persistence on a farrow-to-finish pig farm in which pigs may be co-infected with IMVs [24]. This model gave insights on HEV spread and persistence and evidenced or confirmed several risk factors, e.g. the type of housing for gestating sows, cross-fostering and mingling practices and health status regarding the IMVs. However, this model only explored HEV dynamics in a single and isolated farrow-to-finish herd, without taking into consideration animal trade with other holdings, although pig movements are likely to play a pivotal role in HEV dynamics in the pig production sector. For instance, Nantel-Fortier et al. [25] reported the presence of HEV inside and outside farm buildings, on trucks and in slaughterhouse yards, thus suggesting viral transmission between farms and throughout the production network. Recently, we have also shown, by combining French network indicators with epidemiological data, that the in-degree and ingoing closeness of farms were associated with high HEV within-farm seroprevalence [26].

To represent infection spread at a regional or national scale, multi-scale models can be designed by coupling infection dynamics within herds together with interactions between interconnected herds. Such approaches have already been developed, particularly to explore the transmission of bacterial diseases between cattle farms [27–30] or pig herds [31]. Several approaches have been recently used to implement such models that may be computationally challenging [32–34]. In particular, the SimInf package developed in R software is recognized as an efficient and flexible modelling framework for fast event-based epidemiological simulations of infectious disease spread [32]. It makes it possible to integrate within-herd infection dynamics as a continuous-time Markov process and demographic data as scheduled events. Thus, using the SimInf framework, the aims of our study were: *(i)* to model the spatio-temporal spread of HEV in a cluster of highly connected French pig farms, real pig movement data and HEV within-herd epidemiological dynamics being incorporated; *(ii)* to investigate different introduction and control scenarios.

## 2. Materials and methods

### 2.1 Population dynamics model

#### 2.1.1 Farms’ structure: type, facilities, populations, management system

**Fig 1:**
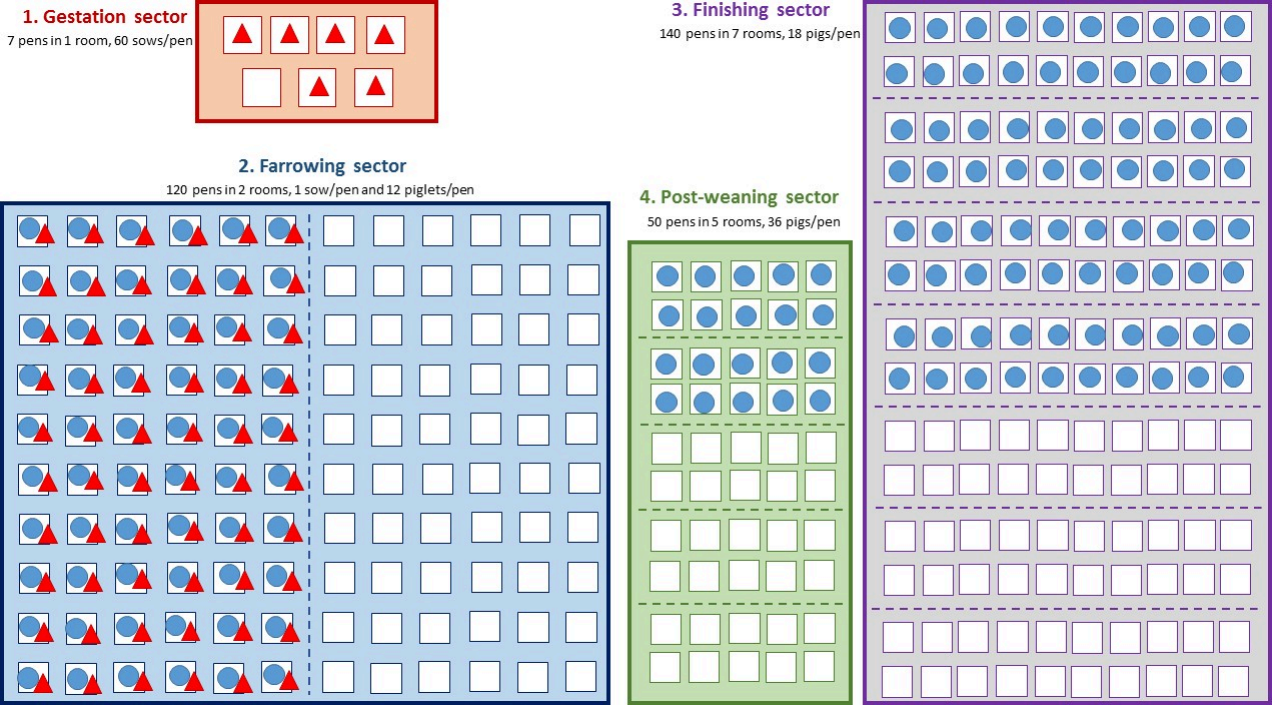
Farm structure, facilities and populations considered. Farms can be composed of one to four sectors depending on their type: gestation, farrowing, post-weaning and finishing sectors (coloured squares). Each sector is divided into rooms (dashed lines), that are composed of pens (white squares). Two populations are considered: breeding sows (red triangles) and growing pigs (blue dots).

Eight farm types are considered: nucleus (*SEL*), multiplication (*MU*), farrow-to-finish (*FF*), farrowing (*FA*), farrowing post-weaning (*FPW*), post-weaning (*PW*), post-weaning finishing (*PWF*) and finishing (*FI*) farms. All farms (within each type) were assumed to have the same structure and size (Fig 1), accounting for one to four sectors, depending on their type (Table 1): gestation, farrowing, post-weaning (i.e. nursery) and finishing sectors. Each of the sectors is divided into rooms, including themselves several pens. Two populations are considered: breeding sows and growing pigs. Depending on its type, a farm can host one or both populations (Table 1).

**Table 1:** Types of sectors, animal populations and events per farm depending on the farm type. Farms are composed of one to four sectors, depending on their type: nucleus (*SEL*), multiplication (*MU*), farrow-to-finish (*FF*), farrowing (*FA*), farrowing post-weaning (*FPW*), post-weaning (*PW*), post-weaning finishing (*PWF*) and finishing (*FI*) farms. They can rear one or two populations (breeding sows, growing pigs). Six types of events can occur depending on the farm type: movement of sows from gestation to farrowing sector (*ges-fa*); piglet birth (*birth*); movement of sows from farrowing back to gestation sector (*fa-ges*); movement of piglets from farrowing to post-weaning sector (*fa-pw*); movement of growing pigs from post-weaning to finishing sector (*pw-fi*); movement of growing pigs leaving the finishing sector (*fi*).

		Farm type							
		SEL	MU	FF	FA	FPW	PW	PWF	FI
**Sectors**	**Gestation**	x	x	x	x	x			
	**Farrowing**	x	x	x	x	x			
	**Post-weaning**	x	x	x		x	x	x	
	**Finishing**	x	x	x				x	x
**Animal populations**	**Breeding sows**	x	x	x	x				
	**Growing pigs**	x	x	x	x	x	x	x	x
**Events**	***ges-fa***	x	x	x	x	x			
	***birth***	x	x	x	x	x			
	***fa-ges***	x	x	x	x	x			
	***fa-pw***	x	x	x	x	x			
	***pw-fi***	x	x	x		x	x	x	
	***Fi***	x	x	x				x	x

Animals evolve in a sequential way through the above-mentioned facilities: the breeding sows in the gestation and farrowing sectors; the growing pigs in the farrowing, post-weaning and finishing sectors. Thus, the two populations physically interact in the farrowing sector only. The farms are managed according to a batch-rearing system (BRS), meaning that the herd population is divided into sets of individuals from the same physiological stage, called batches. For instance, for farms rearing sows, the reproductive cycles of sows belonging to a given batch are synchronised so that all breeding events occur at the same time for all sows. Consequently, a given batch of sows gives birth to piglets simultaneously, these contemporary piglets forming a group of growing pigs also constituting a batch. The batches are managed with an all-in-all-out strategy, i.e. all animals from a batch leave a facility simultaneously and enter an empty room at once. In the model, all farms are considered to be managed with a 7-batch rearing system (i.e. a 3-week interval management system), with parameters being detailed in Table 2.

**Table 2:** Parameters governing the population dynamics model in a 7-batch rearing system. *FA*: farrowing farms, *FPW*: farrowing post-weaning farms, *SEL*: nucleus farms, *MU*: multiplication farms, *FF*: farrow-to-finish farms.

Parameter description (unit)	Value	
Duration of a sow reproductive cycle (days)	142	
- Duration in gestating room (days)	107	
- Duration in farrowing room (days)	35	
Duration of a growing pig cycle (days)	180	
- Duration in farrowing room (days)	28	
- Duration in post-weaning room (days)	86	
- Duration in finishing room (days)	94	
Interval between two successive batches (days)	21	
Annual renewal rate of sow herds (%)	40	
Number of animals:	*In FA and FPW*	*In SEL*, *MU and FF*
- Total number of sows	420	210
- Number of sows per batch	60	30
- Number of piglets per litter	12	
- Number of piglets per batch	720	360

#### 2.1.2 Population dynamics processes

*Life cycle of breeding sows and growing pigs*. After 107 days in the gestation sector (i.e. seven days before farrowing), sows from a batch are transferred into the farrowing sector (one sow per pen) where they give birth to 12 piglets each (Table 2). Dams remain with their litter for four weeks until weaning. At the end of the lactation period, sows are moved back to the gestation sector to begin a new reproductive cycle, when piglets are moved to an empty nursery room (36 pigs per pen, three litters being gathered in one pen). Piglets stay in the nursery sector until 86 days of age when they are moved to a finishing room (18 pigs per pen, i.e. 1.5 litter per pen). When they are 180 day old (i.e. after 94 days in the finishing sector), they are sent to the slaughterhouse. Every 21 days, five replacement gilts are introduced in herds rearing sows and five sows are culled.

**Fig 2:**
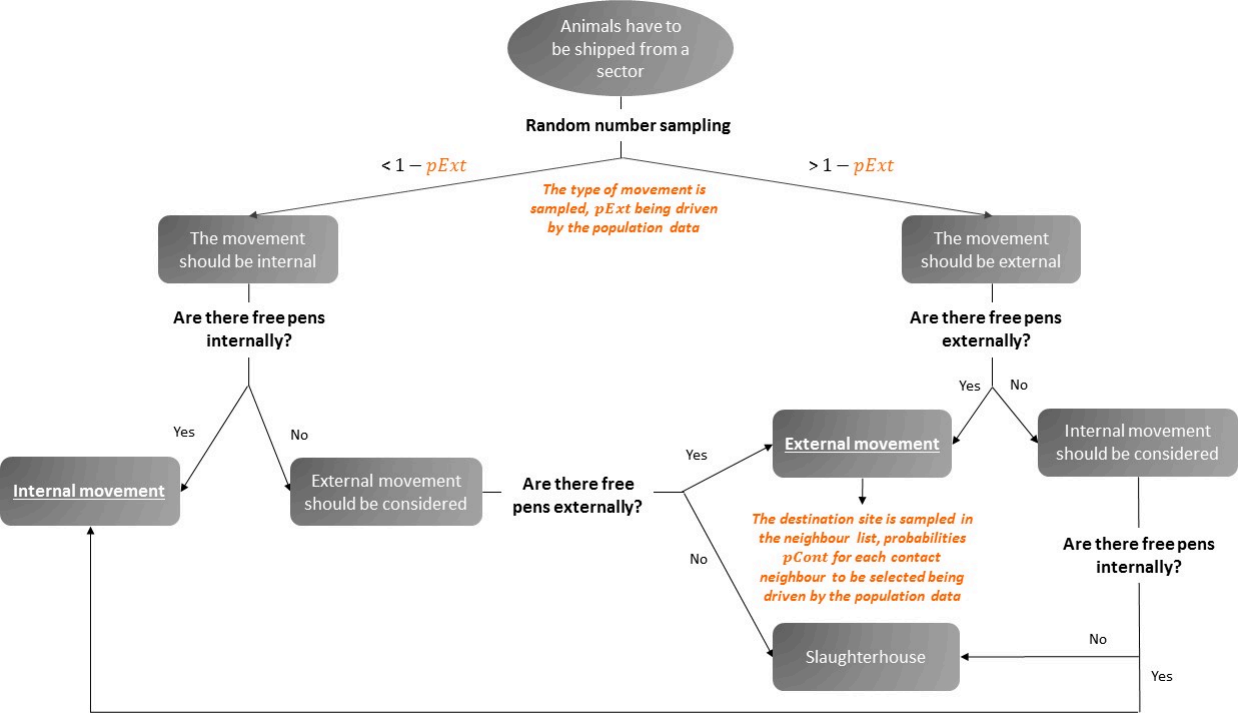
Selection process of the movements’ destinations. Each time animals have to be shipped from a sector, as defined by the production cycle, the type of event (i.e. internal versus external) is determined according to the probability *pExt* that is the probability that animals are shipped externally, as defined by the population data. In cases of no free pens found internally (resp. externally), external (resp. internal) movement is considered. If all pens (internally and in contact farms) are full, animals are sent to slaughterhouse. If animals are shipped externally, the destination site is sampled in the contact neighbours of the farm of origin, the probability *pCont* of a destination farm to be sampled being defined in the population data.

*Implementation of population events*. Six types of events can occur in the population depending on the farm type (Table 1): movement of sows from gestation to farrowing sector (*ges-fa*); piglet birth (*birth*); movement of sows from farrowing back to gestation sector (*fa-ges*); simultaneous movement of piglets from farrowing to post-weaning sector (*fa-pw*); movement of growing pigs from post-weaning to finishing sector (*pw-fi*); movement of growing pigs leaving the finishing sector (*fi*). Event times are determined deterministically by the different cycle durations as explained above. The number of animals to be moved are also fixed by the production system, as described above (Table 2, Fig 1). The three first types of events (corresponding to the sow reproductive cycle: *ges-fa*, *birth*, *fa-ges*) are always internal (i.e. the animals remain in the same farm), when the three others (corresponding to movements of growing pigs: *fa-pw*, *pw-fi*, *fi*) can be either internal or external (i.e. the animals are shipped to another site). Selecting the pens of destination is a two-step process detailed in Fig 2. First, the type of movement (internal or external) is selected with probability *pExt* that the animals are shipped to another farm, derived from real movement data (section 2.1.3). In case of external movement, the destination site is sampled among the set of possible destination farm from the movement database (see below). When leaving the finishing sector (*fi* event), two possible pathways were considered for growing pigs: *(i)* animals leaving *FF*, *PWF* and *FI* farms are sent to the slaughterhouse; *(ii)* a fraction of females is used for the renewal of the sow population either on the same farm (i.e. self-renewal, in *SEL* farms) or on another farm (in cases of animals reared in *SEL* and *MU* farms), and the others are sent to the slaughterhouse. Again, the choice of the destination of finishing events is driven by the population data presented in the following section.

#### 2.1.3 Data on animal movements between farms

**Fig 3:**
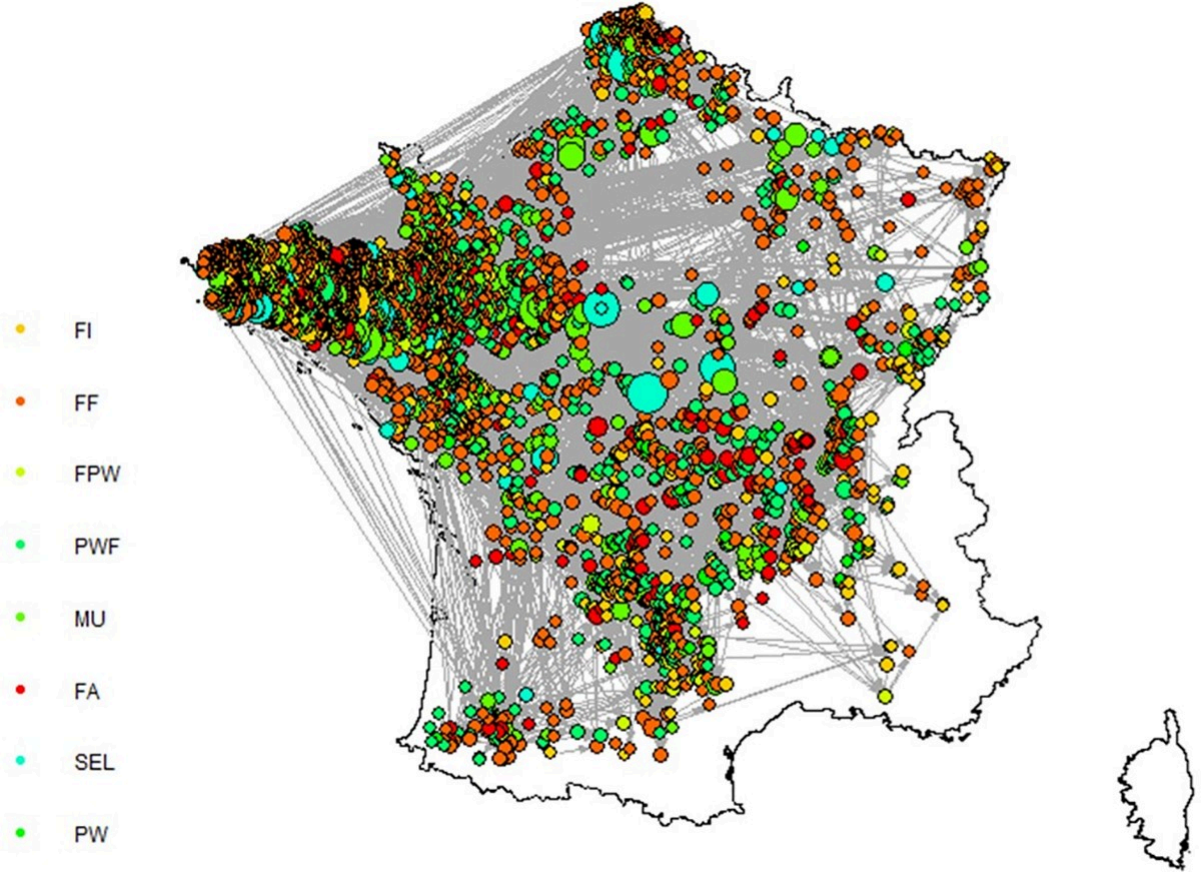
Largest community in the pig movement network in France (2012–2014), derived from Salines et al. [35]. Using Infomap algorithm, a large community including 3,017 farms was identified in the French pig movement network (data from 2012 to 2014). Farm and movement data from this community was used as input population data in the present model. The size of the dots is proportional to the total degree of the holding, the colours are related to the farm type. *FI*: finishing farm, *FF*: farrow-to-finish farm, *FPW*: farrowing post-weaning farm, *PWF*: post-weaning finishing farm, *MU*: multiplication farm, *FA*: farrowing farm, *SEL*: nucleus farm, *PW*: post-weaning farm.

*Dataset*. French pig movement data recorded during the period 1^st^ June 2012 to 31^st^ December 2014 were used to drive the population demographics in the model. The data originated from the National Swine Identification Database (BDporc). The dataset, described in detail in Salines et al. [35], contained 21,446 farms and 2,382,510 between-farm movement records. Briefly, the main features of all swine holdings in mainland France (continental France and Corsica) were included in the database: identification number (ID), type of holding, type of farming activity, farm size and location. Movements of pigs were reported at the batch level with the following information: farm IDs where animals were loaded or unloaded, round number and chronological sequence of the operations forming the round, batch size and animal category. First, as described in Salines et al. [35], a one-mode directed network was built, with holdings being considered as nodes, and movements between two nodes as links. In this network, called Animal Introduction Model in Salines et al. [35], in-between movements forming a round were replaced by direct movements between holdings, i.e. intermediate transit movements of a truck through a farm without unloading any animal were neglected. The analysis of the network revealed the existence of communities, defined as subsets of nodes in which there are significantly more links than expected by chance—i.e. groups of highly connected farms (Infomap algorithm [36]). This approach evidenced a large community including 3,017 farms (Fig 3), among them 55 *SEL*, 210 *MU*, 1,375 *FF*, 86 *FA*, 62 *FPW*, 8 *PW*, 546 *PWF* and 675 *FI* farms. In this community, around 78,000 movements occurred over the study period. Data derived from this community were used to feed SimInf population dynamics sub-model. To achieve this task, we first defined a standard herd size, structure and batch-rearing system to all herds, corresponding to the average characteristics over all the community. Within-farm movements were scheduled following the evolution of the animals through their life- or reproductive-cycles. Who-to-Whom (site-to-site) contact probabilities were then evaluated over the study period to represent the external movements, with a rescaling step to take into account the difference between the standard and the actual herd sizes.

*Calculation of the probability for a movement to be external*. For each farm *i* in the community, the probabilities ${pExt}_{i}^{fa-pw},{pExt}_{i}^{pw-fi}$ and ${pExt}_{i}^{fi}$ that the corresponding possibly external movements (*fa-pw*, *pw-fi* and *fi*, respectively) are actually external have been calculated. For *FA* farms, *fa-pw* movements are always external, so that:

$${pExt}_{i}^{fa-pw}=1$$

Similarly, *pw-fi* movements are always external for *FPW* and *PW* farms, leading to:

$${pExt}_{i}^{pw-fi}=1$$

for these two farm types.

For the other farm types, one may assume that, for an average-sized farm as designed in the population model, the total number of animals shipped over the study period from a sector *a* to a sector *b* is:

$$n_{average}^{a,b}=\frac{n_{days}}{BBI}\times n_{batch}^{pigs}$$

where *n*_*days*_ is the total number of days over the study period, *BBI* the number of days between two successive batches (i.e. between-batch interval) and $n_{batch}^{pigs}$ the average number of pigs per batch.

Denoting $R_{average}^{i}$ the ratio between the actual size of a farm *i* as recorded in the population data and the average size of the farm *i* as designed in the population model, the expected number of animals shipped by the farm *i* from a sector *a* to a sector *b* over the study period can be expressed as:

$${nExp}_{i}^{a,b}=n_{average}^{a,b}\times R_{average}^{i}$$

Let ${nObs}_{i}^{a,b}$ denote the observed number of animals shipped externally by a farm *i* from a sector *a* to the sector *b* of another farm (as recorded in the population data). Then, the probability that the movement from a sector *a* of a farm *i* to a sector *b* is external is:

$${pExt}_{i}^{a,b}=\frac{{nObs}_{i}^{a,b}}{{nExp}_{i}^{a,b}}$$

*Calculation of the contact probability associated to each neighbour*. For each external movement from a sector *a* of a farm *i* to an external sector *b*, the probability that the movement is directed to a contact farm *j* is calculated by:

$${pCont}_{i,j}^{a,b}=\frac{n_{i,j}^{a,b}}{n_{i}^{a}},$$

where $n_{i,j}^{a,b}$ is the number of animals shipped from the sector *a* of the farm *i* to the sector *b* of the contact farm *j* over the study period, as observed in the population data, and $n_{i}^{a}$ is the total number of animals shipped externally from the sector *a* of the farm *i* over the study period, again as observed in the population data.

*Final structure of input data*. Finally, 11 variables were used to describe each of the 3,017 farms and to drive the population dynamics: farm ID, farm type, and nine variables corresponding to the contact matrix with contact probabilities associated to each sector of each farm, as followed:

✓ probability that farrowing to post-weaning movements are external (${pExt}_{i}^{fa-pw}$);
✓ IDs of the contact farms for farrowing to post-weaning movements;
✓ probabilities ${pCont}_{i,j}^{fa-pw}$ that farrowing to post-weaning movement is directed to each of the contact farms;
✓ probability that post-weaning to finishing movements are external (${pExt}_{i}^{pw-fi}$);
✓ IDs of the contact farms for post-weaning to finishing movements;
✓ probabilities ${pCont}_{i,j}^{pw-fi}$ that post-weaning to finishing movement is directed to each of the contact farms;
✓ probability that movements from finishing sector are external (${pExt}_{i}^{fi}$);
✓ IDs of the contact farms for movements from finishing sector;
✓ probabilities ${pCont}_{i,j}^{fi}$ that the movement from finishing sector is directed to each of the contact farms.

### 2.2 Epidemiological model

#### 2.2.1 Epidemiological process

**Fig 4:**
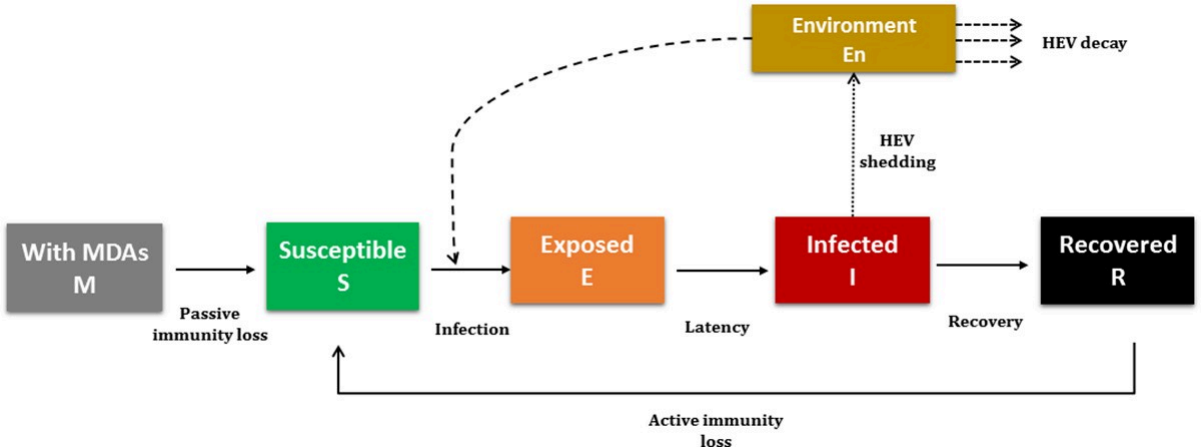
HEV infection process as represented with a MSEIRS model. The epidemiological model has been built as a MSEIR–Maternally Immune (M), Susceptible (S), Exposed (E), Infectious (I) and Recovered (R)–model including an environmental compartment. MDAs: maternally-derived antibodies.

As described in Salines, Rose [24], an MSEIR–Maternally Immune (M), Susceptible (S), Exposed (E), Infectious (I) and Recovered (R)–model including an environmental compartment was considered to describe HEV infection dynamics taking those factors into account (Fig 4). Briefly, new-born piglets born from immune sows acquire anti-HEV maternally-derived antibodies by colostrum intake (health state M), providing complete but temporary protection towards infection. Susceptible (S) pigs can then be infected, entering the exposed (E) state. HEV transmission occurs through faecal-oral route, either by direct contact with an infectious pig or by ingestion of viable virus in the contaminated environment in the pen or the neighbourhood [37, 38]. After the latency period, the infectious animal (I) shed HEV in the environment, where the virus can continue to be viable, feeding the environmental viral pool. Thus, the overall virus load in a pen’s environment corresponds to the accumulation of viral particles shed by all infectious individuals, partially compensated by faeces removal through the slatted floor, the natural decay of the virus and the cleaning/disinfecting operations of empty pens [39]. Recovered pigs (R) lose their immunity over time, assuming a gamma-distribution for antibody waning, and eventually revert to full susceptibility (S). Transitions between epidemiological statuses occur stochastically.

#### 2.2.2 Forces of HEV infection and HEV infection process

As described in Salines et al. [24], HEV force of infection takes two components into account: a within-pen and a between-pen force of infection. Briefly, one infectious pig can infect its pen mates by direct contact or indirectly through its contaminated faeces accumulated in the environment, leading to the following within-pen force of infection:

$$\lambda_{p}^{\mathrm{HEV},\mathrm{wp}}(t)=\frac{\beta_{HEV}\times I_{p}^{HEV}(t)+\beta_{E}^{wp}\times Q_{p}\times Q_{ing}}{N_{p}(t)},$$ (1)

where *N*_*p*_(*t*) and *I*_*p*_ correspond to the total number of animals and the number of infected animals in the pen *p* at the time *t*, respectively. *β*_*HEV*_ denotes the individual HEV transmission rate. $\beta_{E}^{wp}$ is the HEV environmental transmission rate within a pen, corresponding to the average number of animals that can be infected by a single genome equivalent present in the pen environment [22, 39]. *Q*_*ing*_ is the quantity of faeces ingested by a pig per day [38]. *Q*_*p*_ is the HEV quantity accumulated in the pen *p*, calculated as follows:

$$Q_{p}(t)=Q_{p}(t-1)\times (1-\varepsilon_{1})\times (1-\varepsilon_{2})+\frac{w_{HEV}\times I_{p}^{HEV}(t)}{N_{p}(t)},$$ (2)

where *w*_*HEV*_ is the quantity of HEV particles shed in the environment by an infectious pig per gram of faeces. *ε*_1_ and *ε*_2_ are the daily proportion of faeces passing through the slatted floor and the daily HEV mortality rate, respectively. A third decay rate, *ε*_3_, corresponding to the proportion of faeces eliminated through cleaning operations, is sporadically applied when the room is emptied, and the batch is transferred to the next sector.

Moreover, contaminated faeces shed by pigs in a given pen can be transferred to an adjacent pen and are therefore likely to infect a susceptible animal in the adjacent pen. Thus, the between-adjacent-pen force of infection of a pen *p* is equal to the sum of the weighted force of infection of its two neighbours.

$$\lambda_{p}^{HEV,bap}=Q_{ing}\times \beta_{E}^{bap}\times (\frac{Q_{p-1}+Q_{p+1}}{N_{p}}),$$ (3)

where $\beta_{E}^{bap}$ is the HEV indirect environmental transmission rate between pens [39].

Finally, the infection process is event-driven owing to Gillespie algorithm with transition rates as described in Table 3.

**Table 3:** Transition rates for each health state transition as illustrated in Fig 4. *λ* is the global force of infection as described in Eqs (1) and (3), *ρ* is the latency rate for exposed animals E, *γ* is the recovery rate for infectious animals I, *σ* and *μ* denote the maternal and active immunity waning respectively.

Health state transition		Transition rate
Passive immunity waning	M → S	*σ*×*M*
Infection	S → E	$(\lambda_{p}^{\mathrm{HEV},\mathrm{wp}}+\lambda_{p}^{\mathrm{HEV},\mathrm{bap}})\times S$
Latency	E → I	*ρ*×*E*
Recovery	I → R	*γ*×*I*
Active immunity waning	R → S	*μ*×*R*

#### 2.2.3 Epidemiological parameters

All parameters involved in the infectious process are fully described in Table 4 along with their definition and the origin of the input values. Since HEV dynamics has been shown to be strongly affected by co-infections with immunomodulating viruses such as PRRSV or PCV2 [22–24], some epidemiological parameters of the model depend on the farm’s status regarding IMVs.

**Table 4:** Epidemiological parameters governing the HEV infection dynamics in cases of IMV-free or IMV-positive farms. IMV: immunomodulating virus.

Notation	Parameter description (unit)	Value		Reference
		IMV-free farms	IMV-positive farms	
$D_{HEV}^{M}$	Duration of maternal immunity (days)	45		[20]
$D_{HEV}^{E}$	Latency duration (days)	7.4	13.1	[39] [22]
*β*_*HEV*_	Direct transmission rate (pigs/day)	0.15	0.70	
$\beta_{E}^{wp}$	Within-pen environmental transmission rate (g/ge/day)	2.10^−6^	6.6.10^−6^	
$\beta_{E}^{bap}$	Between adjacent pen environmental transmission rate (g/ge/day)	2.10^−8^	6.6.10^−8^	
*w*	Quantity of HEV particles shed in faeces (ge/g/day)	10^4^	10^6^	
*Q*_*ing*_	Average quantity of faeces ingested by a pig (g/day)	25		[38]
*ε*_1_	Faeces elimination rate through slatted floor (/day)	0.70		Expert opinion (ANSES expert group)
*ε*_2_	HEV decay rate in the environment (/day)	0.08		[40]
*ε*_3_	Faeces removal rate by cleaning	0.98		Expert opinion (ANSES expert group)
$D_{HEV}^{I}$	Infectious period (days)	9.7	48.6	[39] [22]
$D_{HEV}^{R}$	Duration of active immunity (days)	185 (Γ(6.2; 30))		Expert opinion (ANSES expert group)

### 2.3 Initialisation and simulations

At the beginning of a simulation, all herds rearing sows (i.e. *SEL*, *MU*, *FF*, *FA* and *FPW*) were composed of seven batches of sows, all being in the susceptible health state; the other farms were empty. At the end of the first year, i.e. after a period of population’s initialisation, one HEV exposed gilt was introduced in a farm when a replacement event happens. The index farm (i.e. the farm in which a positive gilt was introduced) was sampled according to different criteria depending on the scenario tested (see below). We assumed no subsequent introduction of HEV infected animals on the index farm. Simulations were run for five years after HEV introduction. One hundred simulations were run for each tested scenario. For computational reasons, the number of animals in each epidemiological state in every pen of every farm was recorded four times a year.

### 2.4 Assessment of characteristics related to HEV spread in the network and evaluation of potential scenarios

#### 2.4.1 Outcomes

Within-farm HEV dynamics was described by reporting within-herd HEV prevalence in sows and growing pigs on the index farm and HEV on-farm persistence five years post-introduction. Three outcomes were then selected to assess HEV spread in the network and evaluate the risk of HEV introduction into the food chain: *(i)* the proportion of HEV positive farms over the study period, i.e. the proportion of farms having at least one HEV-infected animal; *(ii)* the time at which farms got infected; *(iii)* the proportion of HEV-positive pigs sent to the slaughterhouse over the study period.

#### 2.4.2 Scenarios

Eight different scenarios were run, as described in Table 5 to explore the impact of the type of the farm of introduction (*SEL*, *MU*, *FF* or *FA*) and of decreasing IMV prevalence in the community (going from 100% to 60% of IMV-positive FF farms) on the outcomes.

**Table 5:** Description of the different scenarios (S) of the HEV between-herd model. IMV: immunomodulating virus, *SEL*: nucleus farm, *MU*: multiplication farm, *FF*: farrow-to-finish farm, *FA*: farrowing farm.

Proportion of IMV-free FF farms	Type of the index farm			
	*SEL* with ${pExt}_{i}^{fi}>0.1$	*MU* with ${pExt}_{i}^{fi}>0.1$	*FF* with more than 5 different contacts	*FA* with more than 5 different contacts
**0**	S1	S2	S3	S4
**0.4**	S5	S6	S7	S8

#### 2.4.3 Statistical models

Three statistical models were built:

- A logistic regression was performed to compare the proportion of HEV-infected farms in the community depending on the type of the index farm and on the proportion of IMV-free FF farms in the community.
- A cox-proportional hazard model was used to assess the influence of four variables on farms’ HEV positivity, with the simulation being included as a frailty effect. The four explanatory variables were: *(i)* at the population scale: the type of the index farm and the proportion of IMV-free FF farms; *(ii)* at the individual farm scale: the farm type and the IMV-status (positive or negative). The effect of the interaction between the farm type and the farm IMV-status was also evaluated.
- A generalised estimating equation (GEE) logistic regression was used to compare HEV prevalence in pigs slaughtered in the community depending on the type of the index farm and on the proportion of IMV-free FF farms in the community. The simulation was included as a repeated statement in the model to take into account the non-independence of the proportions of positive pigs for the different farms in a given simulation.

Statistics were performed using SAS 9.1. software (functions *proc logistic*, *proc genmod* and *proc phreg*).

## 3. Results

### 3.1 Descriptive results of the population and epidemiological dynamics

#### 3.1.1 Demographics

At the end of the study period, an average of 406,560 sows and 5,456,799 pigs were present in the community which is consistent with the expected number of pigs on 3,017 farms. A total of 32,629,140 movements occurred over the six years (S1 File). Among them, 15.3% were between-farm movements when the others were within-herd (i.e. between-sector). More precisely, 12.9%, 7.4% of *fa-pw* and *pw-fi* movements were external, respectively.

#### 3.1.2 HEV dynamics on the index farm

After the introduction of an HEV-infected gilt in the gestation sector, an epidemic peak was first observed in the breeding part of the herd due to massive infections of a large pool of naive animals (S2 File). Infected sows entering the farrowing sector then initiated the infectious process in growing pigs by infecting suckling piglets. The latter spread the infection in the nursery and finishing sectors. HEV prevalence levels were lower on *SEL* and *MU* farms than on *FF* and *FA* farms (S2 File).

### 3.2 Factors affecting HEV spread in the community

**Fig 5:**
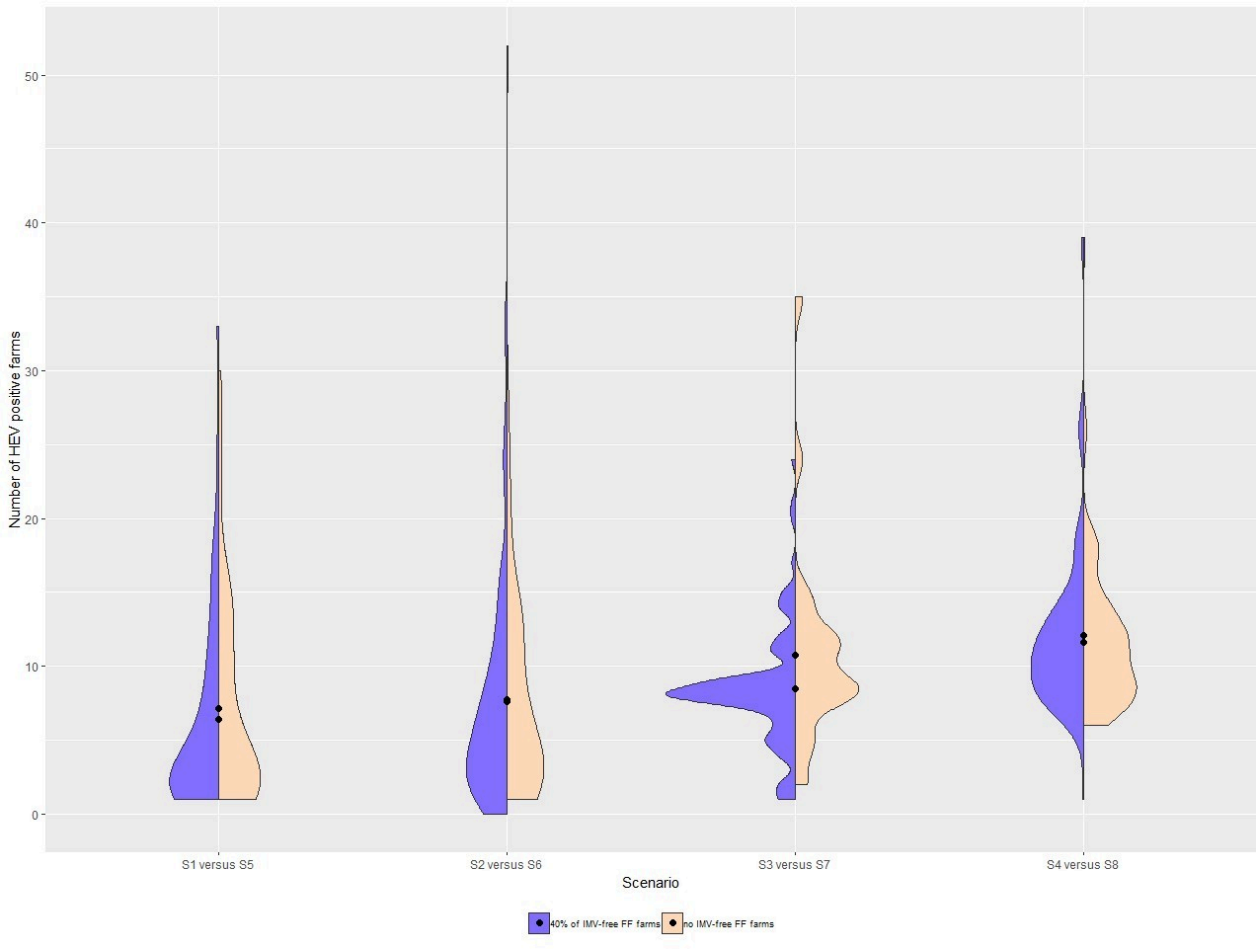
Distribution of the number of HEV positive farms depending on the scenario. S: scenario; FF: farrow-to-finish pig farm.

The distribution of the number of HEV positive farms in the eight tested scenarios is presented in Fig 5. The maximum number of positive farms was 52, with on average nine farms getting infected. In case of *FF* index farm, at least six farms were infected when all FF farms were IMV-positive. The minimal number of infected farms fell to one when the proportion of IMV-positive herds was reduced to 60%.

As shown in Table 6, the proportion of HEV-positive farms over the study period was affected both by the type of the index farm, with a higher proportion of infected farms in case of HEV introduction on a *MU*, *FF*, *FA* farm compared to on a *SEL* farm (Odds Ratio = 1.14 [1.06–1.23], OR = 1.42 [1.33–1.52] and OR = 1.76 [1.65–1.88], respectively), and by the proportion of IMV-free *FF* farms in the community (OR = 0.93 [0.89–0.97] when the prevalence of IMV-positive farms was 60% compared to 100%).

**Table 6:** Effect of the index farm and of the IMV situation in the community on the farm-level prevalence over the study period. Summary statistics obtained thanks to a multivariate logistic regression.

Variable	Modality	Results of the multivariate model	
		Odds Ratio [95% CI]	p-value
**Type of the index farm**		Chi² = 335.58	**p < 0.01**
	**SEL**	-	-
	**MU**	1.14 [1.06–1.23]	p < 0.01
	**FF**	1.42 [1.33–1.52]	p < 0.01
	**FA**	1.76 [1.65–1.88]	p < 0.01
**Proportion of IMV-free FF farms**		Chi² = 10.11	**p < 0.01**
	**0**	-	-
	**0.4**	0.93 [0.89–0.97]	p < 0.01

As shown in Table 7, farms got infected earlier in case of HEV introduction on a *FF* or *FA* farm (Hazard Ratio = 1.49 [1.30–1.71] and HR = 1.75 [1.53–2.00], respectively) compared to an introduction on a *SEL* farm. The farm type was also associated with the time to HEV infection with earlier infection of *PWF* farms compared to the other farm types (HR = 1.25 [1.08–1.45]). The proportion of IMV free farms did not significantly influence the time to infection.

**Table 7:** Effect of population and farm features on the farms’ time to HEV infection. Summary statistics obtained thanks to a cox-proportional hazard model with the simulation being included as a frailty effect.

	Variable	Modality	Results of the multivariate model	
			Hazard Ratio [95% CI]	p-value
**Population features**	**Type of the index farm**		Chi² = 93.41	**p < 0.01**
		**SEL**	-	-
		**MU**	1.05 [0.91–1.21]	p > 0.20
		**FF**	1.49 [1.30–1.71]	p < 0.01
		**FA**	1.75 [1.53–2.00]	p < 0.01
	**Proportion of IMV-free FF farms**		Chi² = 0.39	p > 0.10
		**0**	-	-
		**0.4**	0.97 [0.88–1.07]	p > 0.10
**Farm features**	**Farm type**		Chi² = 2544.42	**p < 0.01**
		**SEL**	-	-
		**MU**	0.60 [0.51–0.70]	p < 0.01
		**FF**	0.22 [0.19–0.25]	p < 0.01
		**FA**	0.83 [0.69–0.99]	p < 0.05
		**FPW**	0.27 [0.21–0.36]	p < 0.01
		**PW**	1.20 [0.85–1.70]	p > 0.20
		**PWF**	1.25 [1.08–1.45]	p < 0.01
		**FI**	0.77 [0.66–0.89]	p < 0.01
	**Farm’s IMV status**		Chi² = 0.15	p > 0.20
		**positive**	-	-
		**negative**	1.02 [0.92–1.13]	p > 0.20

### 3.3 Factors affecting the risk of slaughtering HEV-positive pigs

The type of the index farm was associated with the proportion of HEV-positive pigs slaughtered (p < 0.01). HEV introduction in a *MU*, *FF* or *FA* farm led to a higher risk of having HEV-positive livers entering the food chain compared to the HEV introduction on a nucleus farm (OR = 2.07 [1.69–2.55], OR = 2.23 [1.85–2.70] and OR = 4.41 [3.79–5.28], respectively; Table 8). Reducing the prevalence of IMV-infected *FF* farms was associated with a lower risk of slaughtering HEV-positive pigs (OR = 0.88 [0.79–0.98], Table 8).

**Table 8:** Effect of the type of the index farm and of the IMV situation in the community on the proportion of HEV-positive pigs sent to the slaughterhouse. Summary statistics obtained thanks to a generalised estimating equation (GEE) logistic regression model with the simulation being included as a repeated statement.

Variable	Modality	Results of the multivariate model	
		Odds Ratio [95% CI]	p-value
**Type of the index farm**		Chi² = 375.80	**p < 0.01**
	**SEL**	-	-
	**MU**	2.07 [1.69–2.55]	p < 0.01
	**FF**	2.23 [1.85–2.70]	p < 0.01
	**FA**	4.47 [3.79–5.28]	p < 0.01
**Proportion of IMV-free FF farms**		Chi² = 5.53	**p < 0.05**
	**0**	-	-
	**0.4**	0.88 [0.79–0.98]	p < 0.05

## 4. Discussion and conclusions

Though previous studies have shown the potential role of pig trade in the spread of HEV [25, 26], they did not make it possible to describe HEV diffusion at the territory scale in a dynamic and precise way, or to explain the reasons for HEV spread and persistence in the pig production sector, or to assess the efficacy of HEV control measures in the country. This is the reason why the present study reports on the design of a between-herd HEV model that combines HEV within-farm dynamics with pig trade network. For this model, the chosen level of representation was the pen. Indeed, it made it possible to mimic HEV within-farm dynamics consistently with HEV behaviour described in Salines et al. [24]. Moreover, the pen scale appeared as the most relevant one to represent the within-pen environmental accumulation and transmission of HEV, that has been previously evidenced as a pivotal transmission pathway [39]. HEV epidemiological parameters were estimated from several experimental trials [20, 22, 39]. The majority of them differed according to the animal’s health status regarding the IMV: expanded latency and infectious periods, higher transmission rates for IMV-positive animals than for IMV-negative ones. Nucleus and multiplication farms were considered free from immunomodulating viruses consistently with health situations of these farm types in France (as stated in the health charter of pig producers, available online). All or part of production farms were considered IMV-positive, depending on the scenarios tested. In the case of an IMV-infected farm, the HEV epidemiological parameters were the same for all animals, meaning that all HEV infected animals were considered co-infected with the IMV. By doing so, the frequency of co-infection was over-estimated, as well as all HEV outcomes.

Regarding the population structure, the 3,017 represented farms corresponded to French farms belonging to a single community as described in the analysis of the French network of pig movements [35]. These farms have therefore preferential trade relationships likely to favour spread of pathogens. All farms were composed of a given number of pens, grouped into rooms, themselves grouped into sectors. The farm size was standardized for all farms within a farm type, which is one of the limitations of the model since the size seems to be a risk factor as regards HEV [18, 41–44]; this point would require future improvements to fit real data better. The within-farm demographics was deterministically driven by the time pigs should stay in each sector, related to the batch-management system. Again, the batch-management system was the same for all farms (seven batches, i.e. three weeks interval) which could be upgraded in the future to make it possible to explore the effect of the batch-management system, which was shown to affect HEV on-farm persistence [24]. The between-farm demographics was derived from real data recorded in the national pig movement database from 2012 to 2015. These data were incorporated in the model in the form of a contact matrix with probabilities *(i)* for internal or external transfer *(ii)* and, in the latter case, for transfer to a given neighbour. By doing so, possible temporal evolutions of the pig movement network were not taken into account, but the descriptive analysis we had previously performed showed a stable structure of the network over the study period [35].

When introduced on an IMV-positive *FF* farm, HEV spread in an enzootic way, first in the reproductive herd before affecting piglets and growing pigs. Though the prevalence levels observed in this model were higher than in the within-herd model previously built [24] probably in relation with the co-infection of all animals, the overall HEV behaviour was consistent with the published data [19]. HEV prevalence was lower on *SEL* and *MU* farms compared to *FF* farms, which could be explained by their IMV-free status as described in Salines, Rose [24]. Our analysis showed that the number of contaminated farms in the community over the study period was affected by the type of the index farm, with an introduction on a *MU*, *FF* and *FA* farm being more risky than on a *SEL* farm, with an increasing number of positive farms from *MU* to *FA* index farms. This could be explained *(i)* by the different contact patterns between these four farm types, with *FA* farms sending pigs regularly and at age at which they are likely to be HEV-positive; *(ii)* by their different health status regarding the IMV, with *SEL* and *MU* farms being IMV-free when *FF* and *FA* farms were IMV-positive, thus having a higher HEV prevalence and long-lasting persistence. The influence of IMVs was confirmed by the fact that improving the population health status (i.e. decreasing the prevalence of IMV-positive *FF* farms) led to a reduced number of HEV-positive farms over the study period, which highlights again the role of intercurrent pathogens in the HEV dynamics. An interesting outcome is that the dynamics of HEV spread was affected by the farm type (both the type of the index farm and the type of the infected farm) but not by the IMV-related variables. Indeed, the introduction on a *FF* or on a *FA* farm led to a quicker contamination of other farms, which could again be explained by the riskier contact patterns of these farms. Moreover, all farm types were likely to be infected later, except *PWF* farms which got HEV infected earlier because they are frequent receivers of pigs at a risky age of infection. The non-significant results for *PW* farms was probably related to the lack of statistical power given the low number of *PW* farms in the community (only eight). In addition, if *SEL* farms send animals frequently, they send less animals than *FA*, *PW* and *PWF* farms and at a less risky age regarding HEV, the prevalence being low at late fattening stage. Considered together, these results show that at an individual scale, the farm’s susceptibility to HEV infection was more related to its frequency of animals’ introduction than to its own health situation but that on a collective scale, HEV spread on a breeding community was linked both to the population health status and to the contact patterns. Finally, our analyses evidenced that the risk of slaughtering HEV-positive pigs was related to the type of the index farm, with a 4-times higher risk in the case of introduction on a *FA* farm, and to the population health status, with a lower risk when the prevalence of IMV-positive *FF* farms was decreased.

This model developed at a territory scale, has revealed differences in HEV spatial diffusion patterns related to the introduction pathway, the health status of the pig population, and the type of the exposed farms. If *SEL* and *MU* farms are often considered as the riskiest herds in the pig production sector due to large contact chains, the HEV case highlights that contact patterns have to be considered together with farms’ health status regarding immunomodulating pathogens. It appears therefore essential that *SEL* and *MU* farms preserve their IMV-free status, when production farms implement eradication or control programmes of IMVs. From an operational perspective, two strategies may complementarily help eradicate HEV in a farming community: at farm level, internal and external biosecurity practices need to be improved, as well as control measures of intercurrent pathogens; at global level, pig trade may be restructured in a way that minimise movements from infected to negative farms. Our model can be viewed as an experimental one, with theoretical results that cannot be directly extrapolated to the natural conditions. However, if not relevant from an absolute point of view, they make it possible to compare different scenarios and to identify the riskiest elements. As such, these outcomes can support surveillance strategies by helping target farms having a dense contact network and poor health situation. Our study also gives insight on the HEV diffusion pathway in a HEV-free farming community, which could be structured to provide processing companies with safe livers for the production of raw pork products. Further developments of the model would also make it possible to modify the network structure while simulations are running. This could be particularly useful to simulate trade restriction measures or trade reorganisation, which could occur in the case of the introduction of a regulated disease, an epidemic peak or a modification of the producers’ supply network. Incorporating intermediate loading operations could also make it possible to take into account a possible environmental transmission with trucks acting as mechanical vector. These results could also be used as inputs in other studies, e.g. in a quantitative microbiological risk assessment aiming at assessing the risk of consumers to be exposed to HEV. Finally, designing multi-scale models combining complex within-farm dynamics with animal demographics appears particularly relevant to deal with such multifaceted public health issues. Thus, this kind of research approach should be fostered in the future to have a comprehensive and detailed view of pathogen dynamics on a territory scale and support decision-making.

## Supporting information

S1 File **Simulated network description: number of movements (a) and proportion of external movements (b) per type of movement.** *ges-fa*: movements from the gestation to the farrowing sector; *fa-ges*: movements from the farrowing to the gestation sector; *fa-pw*: movements from the farrowing to the post-weaning sector; *fi*: movements from the finishing sector to the slaughterhouse.
(DOCX)

S2 File **HEV prevalence in sows and growing pigs (median, 50% and 95%) on the index farm in case of HEV introduction on a nucleus (a and b) or farrow-to-finish (c and d) farm (Scenarios S1 and S3).** Pink line: median; dark blue area: 50%; light blue area: 95%; SEL: nucleus farm; FF: farrow-to-finish farm
(DOCX)
