# Supplementary material for: A between-herd data-driven stochastic model to explore the spatio-temporal spread of hepatitis E virus in the French pig production network
Source: PLoS One. 2020 Jul 13;15(7):e0230257. doi: 10.1371/journal.pone.0230257 (PMC7357762; doi:10.1371/journal.pone.0230257)

**Supplementary File 1. Simulated network description: number of movements (a) and proportion of external movements (b) per type of movement**

*ges-fa*: movements from the gestation to the farrowing sector; *fa-ges*: movements from the farrowing to the gestation sector; *fa-pw*: movements from the farrowing to the post-weaning sector; *fi*: movements from the finishing sector to the slaughterhouse.


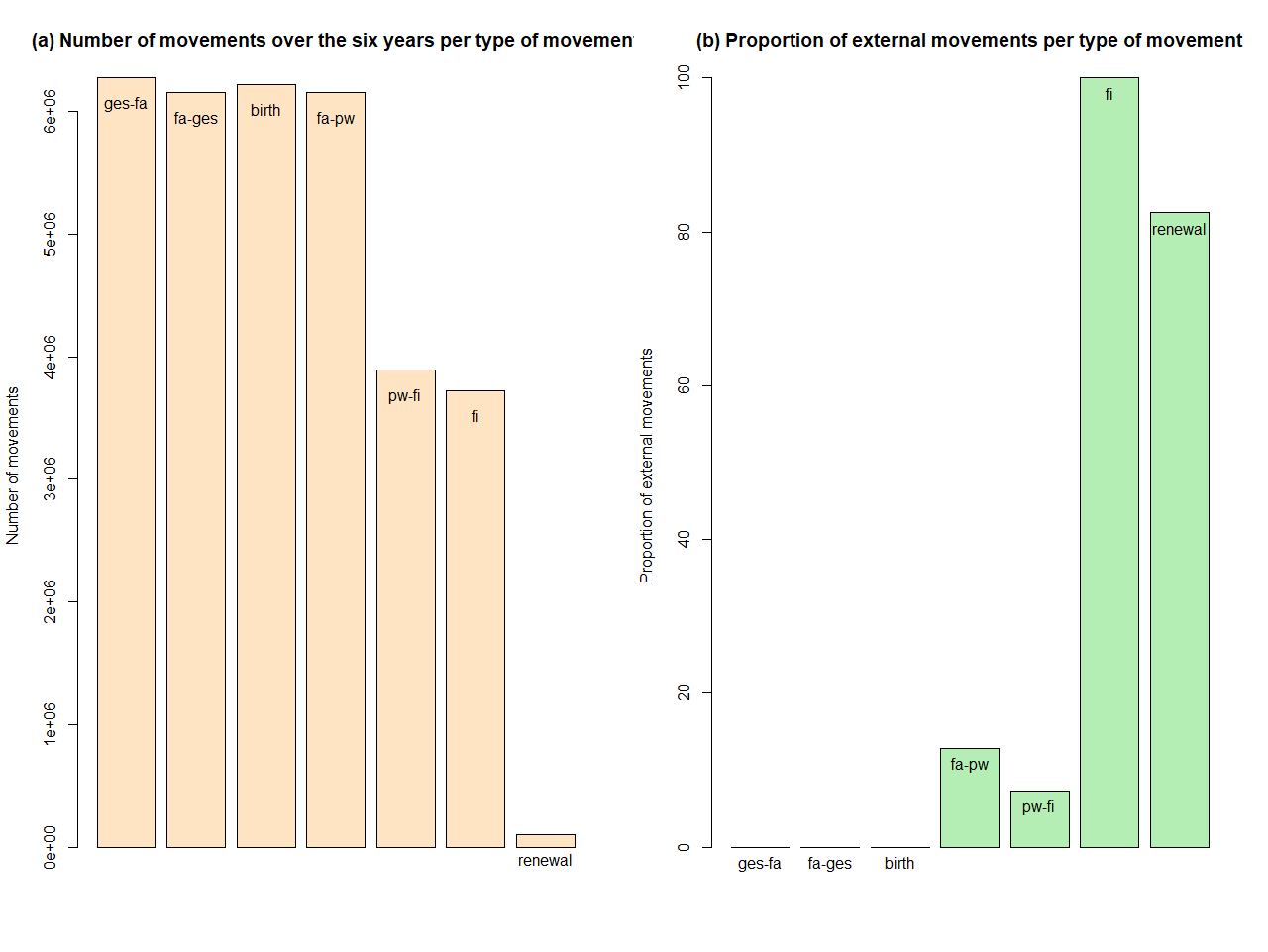

Supplement: S1 File — Simulated network description: number of movements (a) and proportion of external movements (b) per type of movement. ges-fa: movements from the gestation to the farrowing sector; fa-ges: movements from the farrowing to the gestation sector; fa-pw: movements from the farrowing to the post-weaning sector; fi: movements from the finishing sector to the slaughterhouse. (DOCX) [file pone.0230257.s001.docx]
