# Supplementary material for: A between-herd data-driven stochastic model to explore the spatio-temporal spread of hepatitis E virus in the French pig production network
Source: PLoS One. 2020 Jul 13;15(7):e0230257. doi: 10.1371/journal.pone.0230257 (PMC7357762; doi:10.1371/journal.pone.0230257)

**Supplementary File 2. HEV prevalence in sows and growing pigs (median, 50% and 95%) on the index farm in case of HEV introduction on a nucleus (a and b) or farrow-to-finish (c and d) farm (Scenarios S1 and S3).**

Pink line: median; dark blue area: 50%; light blue area: 95%; SEL: nucleus farm; FF: farrow-to-finish farm


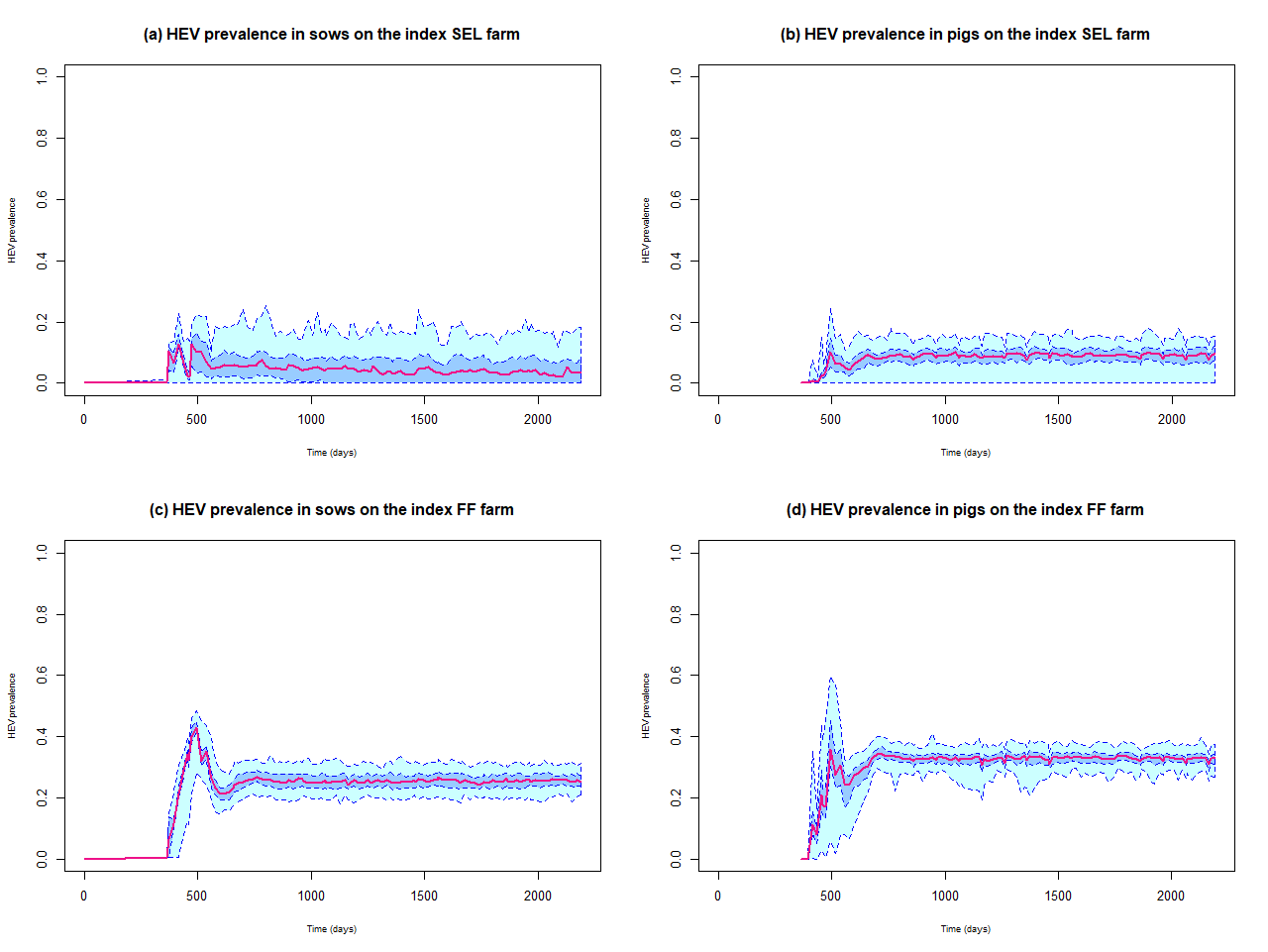

Supplement: S2 File — HEV prevalence in sows and growing pigs (median, 50% and 95%) on the index farm in case of HEV introduction on a nucleus (a and b) or farrow-to-finish (c and d) farm (Scenarios S1 and S3). Pink line: median; dark blue area: 50%; light blue area: 95%; SEL: nucleus farm; FF: farrow-to-finish farm (DOCX) [file pone.0230257.s002.docx]
